# The impact of the 2020 lockdown on the psychological functioning of outpatient psychiatric patients

**DOI:** 10.1192/j.eurpsy.2024.1047

**Published:** 2024-08-27

**Authors:** M. Grah, B. Restek-Petrović, T. Lukačić, V. Grošić, Ž. Milovac, T. Prga Bajić, N. Mayer

**Affiliations:** ^1^University Psychiatric Hospital Sveti Ivan, Zagreb; ^2^Faculty of Dental Medicine and Health, Osijek; ^3^University of Applied Health Sciences Zagreb, Zagreb, Croatia

## Abstract

**Introduction:**

The coronavirus pandemic has led to sudden changes in the lives of people around the world. The health threat, earthquakes and epidemiological measures caused certain psychological reactions in everyone. Psychiatric patients are particularly vulnerable to stress, so we were interested in how the changes at the beginning of the pandemic affected their psychological functioning.

**Objectives:**

To check changes in some areas of psychological functioning of outpatient psychiatric patients after the “lockdown” in 2020 and to examine their connection with some sociodemographic and treatment variables.

**Methods:**

Patients of the University Psychiatric Hospital Sveti Ivan filled out a survey questionnaire designed for the purpose of research, which consisted of sociodemographic data and items examining different areas of psychological functioning, when they arrived for an outpatient check-up.

**Results:**

Variables were formed that examine: changes in unpleasant emotions, lack of support, lack of social interaction, changes in performing daily duties, changes in self-help behaviors and health concerns. Statistical analysis showed a significant increase in all variables, with the largest occurring in lack of social interaction, health concerns, and unpleasant emotions. The predictors of changes in psychological functioning were female gender, younger age in combination with cohabitation with parents, and the number of hospitalizations.

**Conclusions:**

After the “lockdown” in 2020, psychiatric patients report a deterioration in psychological functioning.

**Disclosure of Interest:**

None Declared

